# Frequency of adverse feto-maternal outcomes in patient with placental abruption

**DOI:** 10.12669/pjms.42.1.12261

**Published:** 2026-01

**Authors:** Uzma Kausar, Saima Khattak

**Affiliations:** 1 Uzma Kausar, Medical Officer, Department of Obstetrics and Gynaecology, Medical Teaching Institute Lady Reading Hospital, Peshawar, Pakistan; 2 Saima Khattak, Assistant Professor, Department of Obstetrics and Gynaecology, Medical Teaching Institute Lady Reading Hospital, Peshawar, Pakistan

**Keywords:** APGAR, Feto-Maternal Outcomes, Placenta Abruption, Pregnancy

## Abstract

**Objective:**

This study aimed to determine the frequency of adverse feto-maternal outcomes in patients with placental abruption.

**Methodology:**

This descriptive case series was a study carried out in the Department of Obstetrics and Gynecology, Lady Reading Hospital, Peshawar between January 26, 2022 and July 26, 2022. The sample size of the study was 136 patients who had placental abruption, who were aged between 18 and 40 years, and were more than 24 weeks of gestation and any parity. The feto-maternal outcomes of these patients were compared.

**Results:**

The mean age of the participants was 27.65 ± 6.27 years. The adverse maternal outcomes were acute tubular necrosis (8.8%), disseminated intravascular coagulation (19.1%), postpartum hemorrhage (27.9%), and maternal admission to ICU was detected in 7 (5.1%) patients. The maternal age was significant as associated with DIC (p < 0.05). Adverse fetal outcomes included intrauterine fetal death in 6 (4.4%), preterm birth in 30 (22.1%), low APGAR score in 33 (24.3%), stillbirth in 28 (20.6%), and admission to NICU in 29 (21.3%). The maternal demographic variables were significantly related to preterm birth (p = 0.03) and stillbirth (p < 0.05).

**Conclusion:**

Our study identified the frequency of adverse fetal and maternal outcomes in patients with placental abruption. The fetal outcomes were intrauterine fetal death, pre-term births, low APGAR score, and stillbirth. The acute tubular necrosis, disseminated intravascular coagulation and postpartum hemorrhage were maternal outcomes.

## INTRODUCTION

Placental abruption is defined as the premature separation of a normally implanted placenta from the uterine wall, typically occurring after 20 weeks of gestation.^1^ It affects 0.4% to 1.0% of pregnancies and is a leading cause of maternal morbidity and adverse neonatal outcomes.^2^ The incidence of placental abruption varies across different regions and healthcare systems, ranging from 0.48% to 0.99% in recent studies. A tertiary center in Nigeria reported a prevalence of 0.48%, while studies from India and Poland reported 0.95% and 0.7%, respectively.^3^-^5^ A study from Kolkata, India, found an incidence of 0.99%, and data from Dubai reported 0.61%.^6^,^7^ A systematic review estimated the global incidence of preterm placental abruption at 0.83%.^8^

Common maternal risk factors for placental abruption include hypertension, preeclampsia, diabetes, uterine anomalies, prior cesarean sections, and multiple gestation.^4^,^5^,^7^ Placental abruption (PA) may be complete or partial, and either marginal or central, with clinical implications varying accordingly. Pregnancies complicated by PA are associated with significantly lower gestational age at delivery, increased rates of preterm birth, and low birth weight.^9^ Additional neonatal complications include low Apgar scores, cerebral palsy, hypoxic-ischemic encephalopathy, intracranial hemorrhage, respiratory distress, prolonged hospital stays, and higher NICU admissions. Although maternal mortality is rare, likely due to timely interventions, serious maternal complications such as postpartum hemorrhage, anemia, uterine rupture, HELLP syndrome, and increased blood loss remain prevalent.^4^-^7^

Placental abruption cannot be definitively diagnosed through laboratory tests or imaging, but several evaluations help in ruling out differential diagnoses and guiding management.^10^ Ultrasound is useful for identifying placental location and excluding placenta previa, though its sensitivity for detecting abruption is low especially in acute cases where hemorrhage may be isoechoic and mimic normal placental tissue. In cases managed conservatively, a biophysical profile can help assess fetal well-being, with scores ≤6 suggesting compromise. Baseline investigations such as complete blood count (CBC), coagulation profile (including fibrinogen and PT/aPTT), and blood urea nitrogen (BUN) are essential for maternal assessment. Early blood typing and Rh status should be done in case of transfusion. The Kleihauer-Betke test, while not diagnostic for abruption, quantifies fetal red cells in maternal blood and is critical for Rh-negative mothers, guiding the need for Rh(D) immune globulin to prevent alloimmunization.^10^

To address the lack of comprehensive analysis in previous studies, we aimed to establish the prevalence of adverse feto-maternal outcomes in patients with placenta abruption, with proper control of the possible confounding variables.

## METHODOLOGY

This descriptive case series was conducted during the period of January 26, 2022, to July 26, 2022, at the Department of Obstetrics and Gynecology, Lady Reading Hospital, Peshawar. The sample size of the study consisted of 136 patients, calculated by the WHO sample size calculator and the confidence level of 95% and the margin of error of 4%. The non-probability consecutive sampling was used.

### Inclusion & Exclusion Criteria:

Inclusion criteria included women aged between 18 and 40 years with singleton pregnancies who were in ultrasound, gestation age of over 24 weeks and any parity and diagnosed with placental abruption. The exclusion criteria were placement previa, history of urinary tract infection and disorders associated with bleeding.

The data collection involved obtaining baseline demographic information, including age, gestational age, and parity, with informed consent from patients ensuring confidentiality and safety. Patients were observed until delivery and adverse fetal outcomes, including acute tubular necrosis, disseminated intravascular coagulation, postpartum bleeding, maternal admission to ICU, intrauterine fetal death, preterm birth, low Apgar score, stillbirth, and NICU admission, were documented on a specially designed proforma.

Statistical analysis was performed using IBM-SPSS 23, presenting categorical variables with frequencies and percentages and quantitative variables with mean and standard deviation. Fetomaternal outcomes were stratified by age, gestational age, and parity, with post-stratification Chi-Square Test was applied, p ≤0.05 was considered statistically significant.

### Ethical approval:

The ethical approval was obtained from the Lady Reading Hospital-MTI ethical review board [Reference # 201/LRH/MTI; Dated December 6, 2021].

## RESULTS

**Fig.1:**
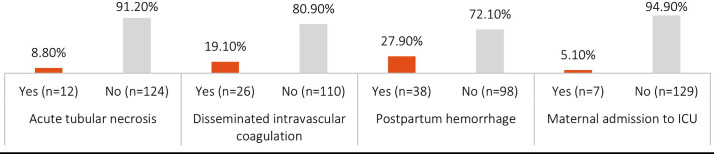
Maternal Outcomes.

This study enrolled 136 pregnant women diagnosed with placental abruption. The patients had a mean age of 27.65 years (±6.27) and a mean gestational age of 32.59 weeks (±4.79). The mean parity among the participants was 2.23±1.04. In terms of age distribution, 88 (64.7%) participants fell within the age group of 18 to 30 years, while 48 (35.3%) were in the age group of 31 to 40 years. The maternal outcomes observed in the study participants is presented in Fig.1. Acute tubular necrosis was observed in 8.8% of cases, while DIC occurred in 19.1%. Postpartum hemorrhage was reported in 27.9% of cases, and 5.1% of participants required admission to the intensive care unit (ICU).

**Fig.2:**
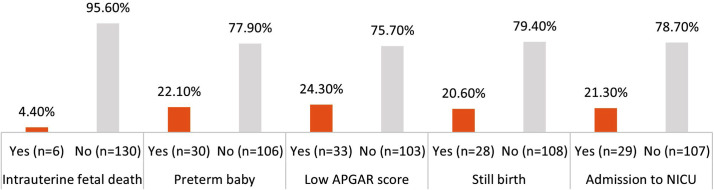
Fetal Outcomes.

Among fetal outcomes, 4.4% (n=6) experienced intrauterine fetal death, 22.1% (n=30) resulted in preterm birth, and 20.6% (n=28) ended in stillbirth. Additionally, 24.3% (n=33) had a low APGAR score, and 21.3% (n=29) required admission to the neonatal intensive care unit (NICU), as illustrated in Fig.2.

### Association of Maternal Outcomes with Maternal Age, Gestational Age and Parity:

Maternal outcomes were examined statistically to analyze outcomes in two age groups: 18 to 30 years and 31 to 40 years, across different gestational age groups: 25 to 31 weeks, 32 to 37 weeks, and >37 weeks, and across different parity groups: 1 to 3 and >3. Chi-square tests were used to evaluate the statistical significance of the observed differences in outcomes across these demographic categories. The frequencies and percentages of each maternal outcome were compared, as illustrated in Table-I. For acute tubular necrosis, postpartum hemorrhage, there was no statistically significant difference between the two age groups (P = 0.43, P= 0.52 respectively). However, for disseminated intravascular coagulation, there was a significant difference observed (P < 0.05), with a higher percentage of cases occurring in the 18 to 30 years age group compared to the 31 to 40 years age group. Additionally, while there was a higher percentage of maternal admissions to the ICU in the 18 to 30 years age group was higher than that in 31 to 40 years age group but the difference wasn’t significant (P = 0.23). There were no statistically significant differences among the gestational age groups in the case of acute tubular necrosis, postpartum bleeding, and maternal ICU hospitalization (P > 0.05). Similarly, for disseminated intravascular coagulation, there was no significant difference in occurrence among the gestational age groups (P = 0.81). Acute tubular necrosis, disseminated intravascular coagulation, postpartum hemorrhage, and maternal admission to the ICU, there was no statistically significant differences across the parity groups (P > 0.05).

**Table-I:** Association of Maternal Outcomes with Demographic Variables.

Maternal Outcome		Age		p-value	GA			p-value	Parity		p-value
		18–30	31–40		25–31 weeks	32–37 weeks	>37 weeks		1–3	>3	
Acute Tubular Necrosis	Yes	9 (75.0%)	3 (25.0%)	NS	3 (25.0%)	5 (41.7%)	4 (33.3%)	NS	9 (75.0%)	3 (25.0%)	NS
	No	79 (63.7%)	45 (36.3%)		57 (46.0%)	43 (34.7%)	24 (19.4%)		107 (86.3%)	17 (13.7%)	
DIC	Yes	21 (80.8%)	5 (19.2%)	NS	10 (38.5%)	10 (38.5%)	6 (23.0%)	NS	23 (88.5%)	3 (11.5%)	<0.05*
	No	67 (60.9%)	43 (39.1%)		50 (45.5%)	38 (34.5%)	22 (20.0%)		93 (84.5%)	17 (15.5%)	
Postpartum Hemorrhage	Yes	23 (60.5%)	15 (39.5%)	NS	20 (52.6%)	13 (34.2%)	5 (13.2%)	NS	33 (86.8%)	5 (13.2%)	NS
	No	65 (66.3%)	33 (33.7%)		40 (40.8%)	35 (35.7%)	23 (23.5%)		83 (84.7%)	15 (15.3%)	
Maternal ICU Admission	Yes	6 (85.7%)	1 (14.3%)	NS	2 (28.6%)	3 (42.9%)	2 (28.6%)	NS	5 (71.4%)	2 (28.6%)	NS
	No	82 (63.6%)	47 (36.4%)		58 (45.0%)	45 (34.9%)	26 (20.2%)		111 (86.0%)	18 (14.0%)	

* p<0.05 is considered significant.

### Association of Fetal outcomes with Maternal Age, Gestational Age and Parity:

Fetal outcomes were analyzed using Chi-square test across different age distributions (18 to 30 and 31 to 40 years), gestational age (25 to 31 weeks, 32 to 37 weeks, and >37 weeks), and different parity groups (1 to 3 and >3) to determine statistical significance, presented in Table-II. For intrauterine fetal death, preterm birth, low APGAR score, stillbirth, and admission to NICU, no statistically significant differences were observed using Chi-square test across the age groups (P > 0.05), except for preterm birth (P = 0.006). The statistical significant result shows that preterm birth was highly prevalent in the age range of 31 to 40 years than the age range of 18 to 30 years. The preterm birth was significantly different (P = 0.03) as it was more common in the earlier gestational period. Stillbirth was also significantly different (P < 0.05) with increased incidences in the intermediate gestational age category. No significant difference was found in other outcomes (intrauterine fetal death, low APGAR score and admission to NICU) between gestational age groups (P > 0.05),occurrence of intrauterine fetal death, preterm birth, low APGAR score, stillbirth, or admission to NICU across the two parity groups (P > 0.05).

**Table-II:** Association of Fetal Outcomes with Demographic Variables.

Fetal Outcome		Age		p-value	GA			p-value	Parity		p-value
		18–30	31–40		25–31 weeks	32–37 weeks	>37 weeks		1–3	>3	
Intrauterine Fetal Death	Yes	2 (33.3%)	4 (66.7%)	NS	2 (33.3%)	3 (50.0%)	1 (16.7%)	NS	4 (66.7%)	2 (33.3%)	NS
	No	86 (66.2%)	44 (33.8%)		58 (44.6%)	45 (34.6%)	27 (20.8%)		112 (86.2%)	18 (13.8%)	
Preterm Birth	Yes	13 (43.3%)	17 (56.7%)	NS	16 (53.3%)	13 (43.3%)	1 (3.3%)	NS	25 (83.3%)	5 (16.7%)	<0.05*
	No	75 (70.8%)	31 (29.2%)		44 (41.5%)	35 (33.0%)	27 (25.5%)		91 (85.8%)	15 (14.2%)	
Low APGAR Score	Yes	19 (57.6%)	14 (42.4%)	NS	10 (30.3%)	13 (39.4%)	10 (30.3%)	NS	27 (81.8%)	6 (18.2%)	NS
	No	69 (67.0%)	34 (33.0%)		50 (48.5%)	35 (34.0%)	18 (17.5%)		89 (86.4%)	14 (13.6%)	
Stillbirth	Yes	16 (57.1%)	12 (42.9%)	NS	7 (25.0%)	12 (42.9%)	9 (32.1%)	NS	23 (82.1%)	5 (17.9%)	<0.05*
	No	72 (66.7%)	36 (33.3%)		53 (49.1%)	36 (33.3%)	19 (17.6%)		93 (86.1%)	15 (13.9%)	
NICU Admission	Yes	16 (55.2%)	13 (44.8%)	NS	13 (44.8%)	12 (41.4%)	4 (13.8%)	NS	26 (89.7%)	3 (10.3%)	NS
	No	72 (67.3%)	35 (32.7%)		47 (43.9%)	36 (33.6%)	24 (22.4%)		90 (84.1%)	17 (15.9%)	

* p<0.05 is considered significant.

## DISCUSSION

Our results show that maternal and fetal complications are highly dangerous, which supports the severity of this obstetric emergency. Postpartum hemorrhage (27.9%), disseminated intravascular coagulation (19.1%), and acute tubular necrosis (8.8%) were the most common maternal outcomes, and 5.1% were hospitalized in the ICU. Among fetal outcomes, stillbirth (20.6%), low APGAR (24.3%), preterm birth (22.1%), and NICU admission (21.3%) were common. These statistics are close to other past studies, including Hanchate et al., who also found high prevalence of postpartum bleeding, disseminated intravascular coagulation, and acute renal failure.^11^ Fetal outcomes tend to be poor, too, with high rates of preterm birth, low birth weight, and infant death.^7^,^12^

Importantly, our study adds value by identifying specific demographic associations that have clear clinical implications. We found a statistically significant association between gestational age and both preterm birth (p = 0.03) and stillbirth (p = 0.05). This suggests that earlier gestational stages are particularly at risk for poor fetal outcomes. Additionally, we identified a significant link between maternal age and DIC (p = 0.05), which means that the young women were more likely to be affected by coagulation disorders and that it is necessary to monitor them individually. Parity, on the other hand, did not in our cohort show significant relationships with either the maternal or fetal outcome, as compared to studies like Lee et al., which showed an increased risk of placental abruption in multiparity.^13^ This shows that placental abruption is multifactorial and unpredictable, which emphasizes the necessity of universal preparedness in obstetric care.

Current literature underlines the idea that high age of mothers (>35 years) is an independent factor that determines the risks of adverse obstetric and perinatal outcomes.^14^-^16^ Specifically in the case of placental abruption, a reduced gestational age at birth has consistently been found to be the most potent predictor of adverse neonatal outcomes, particularly in African American mothers.^14^,^17^-^20^ Maternal complications, like the HELLP syndrome and substance-related outcomes, are also found to be more common in this population. Based on these results, healthcare providers ought to focus on preconception counseling and risk-based antenatal care to women of advanced maternal age to reduce the burden of adverse events.

By estimating the prevalence of unfavorable feto-maternal events, as well as analyzing their relationship with maternal age, gestational age, and parity, this research contributes to the current body of knowledge on placental abruption by providing valuable, locally-specific information. Interestingly, preterm births were much higher among the 31-40 years old group, and stillbirths were higher in the 32-37-week gestational group results that highlight the necessity of specific surveillance in these high-risk groups. Other clinically important maternal outcomes that were identified in the study include postpartum hemorrhage (27.9%), DIC (19.1%), NICU admissions (21.3%), and acute tubular necrosis (8.8%) which is an especially underreported maternal outcome. The strong point of the study is that the stratified analysis of the outcome adds more clinical implications to the study method as risk specific patterns are revealed. Nevertheless, the small sample size, the single-center design, and the lack of control over confounding factors (e.g., the status of booking, smoking, socioeconomic background, etc.) can have an impact on generalizability. Further studies are also needed as future work that incorporates multi-centre studies using larger sample size and in a wider range of variables to hone the strategies of risk prediction and management. Comprehensively, our results underscore the clinical significance of early diagnosis, prompt treatment, and personalized attention to the lessening of the burden of feto-maternal complication in placental abruption.

### Limitations:

Although this study offers valuable information, it should be noted that there are several limitations to it. The fact that the sample size is relatively small can be viewed as one of the causes of the differences in outcomes across demographic categories. The study was single center based and it might not be generalizable to larger populations. Although some demographic variables such as maternal age, gestational age and parity seem to have correlation with certain outcomes such as disseminated intravascular coagulation, their general effect on both maternal and fetal outcomes has not been clear. More studies should be conducted to explain the relationship between these variables and negative consequences of cases of placental abruption.

## CONCLUSION

This study contributes to our understanding of the demographic factors influencing maternal and fetal outcomes in cases of placental abruption. The fetal outcomes were intrauterine fetal death, preterm birth, low APGAR score and stillbirth. Acute tubular necrosis, disseminated intravascular coagulation and postpartum hemorrhage were the maternal outcomes. Our results reflect that extensive prenatal care, early diagnosis, and close attention to risk factors are required to reduce the negative impact on both the health of the mothers and the babies. High-risk pregnancy antenatal follow-up and early intervention can greatly lower morbidity associated with it.
